# Role of a School Nurse in the Management of Type 1 Diabetes in Schools: Teachers' Perspectives

**DOI:** 10.7759/cureus.81526

**Published:** 2025-03-31

**Authors:** Despoina Chatziklonidou, Spyridon Rigatos, Evangelos C Fradelos, Aikaterini Toska, Maria Saridi, Anastasios Tzenalis, Ioannis Koutelekos, Eleni N Albani

**Affiliations:** 1 Primary Healthcare, Faculty of Medicine, University of Thessaly, Larissa, GRC; 2 Department of Nursing, University of Patras, Patras, GRC; 3 Laboratory of Clinical Nursing, Department of Nursing, University of Thessaly, Larissa, GRC; 4 Nursing, University of West Attica, Athens, GRC

**Keywords:** management of type 1 diabetes mellitus, management of type i diabetes mellitus, school nurse, school students' health, student with type i diabetes mellitus, type 1 diabetes mellitus, type i diabetes mellitus

## Abstract

Introduction: Type 1 diabetes mellitus (T1DM) is a chronic metabolic disorder and carries the risk of causing serious complications and/or death in the patient. In the school environment, diabetic students need monitoring and support to ensure their health. Teacher perceptions can impact T1D management in schools by influencing their preparedness, support strategies, and response to emergencies. The aim of this study was to assess attitudes and perceptions of teachers regarding the role of a school nurse in the management of students with T1DM in the school environment.

Methods: This qualitative study was conducted in two primary schools with a final sample of 10 teachers. Data were collected through semi-structured interviews using an 11-item open-ended questionnaire. Ethical considerations ensured confidentiality and voluntary participation. Thematic analysis was employed to categorize responses into conceptual units, following an inductive approach. This method, chosen for its strength in capturing the complexity of teachers' experiences and perceptions, provided an in-depth exploration of their perspectives on the role of school nurses in managing students with T1DM.

Results: Three main themes emerged from the thematic analysis. The first theme was Knowledge and Understanding of Type 1 Diabetes Mellitus, the second was Knowledge and Perceptions of Teachers Regarding the Role of the School Nurse in Supporting Students With Diabetes, and the third was Collaboration and Interaction Levels Between Teachers and the School Nurse.

Conclusion: The study concludes that school nurses play a key role in supporting students with diabetes, contributing to their equal inclusion in the school environment. The findings suggest that enhanced collaboration between teachers and nurses, combined with targeted teacher training, can promote a holistic and supportive approach to student care, improving their school life and overall well-being.

## Introduction

Type 1 diabetes mellitus (T1DM) is a chronic metabolic disorder, with diagnosis typically occurring during childhood and adolescence, leading to various challenges for young patients. The complete absence of hormone insulin requires exogenous administration and continuous monitoring of the patient’s blood sugar levels to avoid severe complications, which could even result in death [1,2]. Given the lifelong nature of the disease, individuals with T1DM must develop a structured routine to manage their condition, balancing medical needs with daily activities, including school attendance. The need for strict glycemic control often imposes significant physical and emotional burdens on these young patients, making a supportive school environment essential to their overall well-being and academic success.

A key aspect of the daily life of this age group is attending school, which necessitates their presence in the classroom for a significant part of the day. However, the long hours spent within the school environment complicate the monitoring of their health and increase the risk of potential complications [3,4]. At the same time, significant barriers regarding their uninterrupted participation in school activities arise, as attention and guidance are required during involvement in sports, walks, and school trips [5]. Students with type 1 diabetes often experience concerns about hypoglycemia, which can occur suddenly and require immediate intervention. Such fears can negatively impact their ability to engage in classroom learning and extracurricular activities, reinforcing the need for proper education and preparedness among school staff.

Managing students with type 1 diabetes mellitus within the school community is crucial, with the aim of integrating them as active and equal members of the classroom, respecting their specific needs. School staff play a vital role in fostering a safe, supportive, and engaging learning environment. Their responsibilities extend beyond teaching, encompassing student well-being, conflict resolution, and emergency preparedness. Effective collaboration among educators, administrators, and support staff ensures a positive school culture that nurtures academic success and personal growth [6]. Ensuring their full participation in academic and social activities requires an inclusive approach that prioritizes both their medical needs and emotional well-being. In this context, the role of a school nurse is essential, as they are responsible for monitoring and supporting the diabetic student, working to create a friendly and safe environment for them in the school setting [7,8]. Beyond offering medical support, the school nurse plays a vital role in educating school staff and fellow students about diabetes management, fostering a culture of awareness and inclusivity.

To effectively address the multifaceted role of the school nurse, collaboration with the educational community is paramount, focusing on a holistic approach and ensuring the protection and well-being of the diabetic child within the school environment [9]. This collaboration extends beyond routine medical supervision, involving structured communication between nurses, teachers, parents, and healthcare providers. Schools that implement well-defined policies for diabetes care and provide training for their staff create a safer and more supportive environment for students with type 1 diabetes mellitus. Additionally, peer education programs can further enhance inclusivity and reduce stigmatization, allowing students with diabetes to feel more accepted and understood by their classmates.

Lacking basic knowledge of how to manage emerging health issues and the potential complications they may cause, teachers are unprepared to deal with the potential risks of chronic and serious diseases in their students. Many educators may feel uncertain or anxious when confronted with a diabetes-related emergency, underlining the importance of specialized training. Recognizing the value of active participation of all children in the learning process, the educational community is turning to the provision of specialist support through employment within the school unit of staff suitably qualified to deal with the serious health issues that may arise [3,10]. When teachers receive adequate training on diabetes management, they are more likely to respond confidently and effectively to students’ needs, minimizing disruptions in the classroom and reducing the anxiety associated with medical incidents.

In the context of ensuring the achievement of the important role of school nurses, the collaborative action between them and the school teachers reveals essential value, as through it the quality of their relationships is determined, and therefore, the cultivation of an environment capable of supporting the specific students is promoted [11,12]. Developing protocols for teacher-nurse collaboration can significantly improve the overall management of T1DM in the school setting, creating a structured and consistent approach to student care. Moreover, fostering open communication between teachers, school nurses, and families ensures that any changes in a student’s condition are promptly addressed, preventing potential medical emergencies.

It therefore seems interesting to assess the perceptions and experiences of teachers regarding the presence of the school nurse within the school setting, with the protection of all students as a key concern. By prioritizing the needs of students with type 1 diabetes mellitus and strengthening collaboration between school personnel, a safer and more inclusive learning environment can be achieved.

## Materials and methods

Study design and sample

This qualitative study was conducted in two elementary education schools with student populations of 240 and 200, respectively. The first school employed 20 permanent teachers, while the second employed 15, with the total teaching staff of each school approximating 30. Initially, 13 teachers agreed to participate in the study. However, three withdrew due to time constraints or unfamiliarity with the subject, leaving a final sample of 10 participants [13]. A smaller sample size is consistent with qualitative research methodology as it ensures an in-depth exploration of participants’ subjective perspectives. We employed a purposive sampling approach to ensure the selection of participants with characteristics relevant to the study's objectives, thereby facilitating an in-depth exploration of the research topic.

Data collection techniques

Data was collected through semi-structured interviews using 11-item open-ended questions (Appendix 1). This format allowed for a dialogical process between the interviewer and participants, ensuring a thorough exploration of their perceptions. The semi-structured interview method provided flexibility in narrative exploration, making it ideal for qualitative studies [14]. Open-ended questions were prioritized as they facilitated richer, more detailed responses compared to closed-ended questions, which tend to limit response variability [15,16].

The interview questions aimed to examine the teachers’ attitudes, opinions, and experiences regarding the role and value of a school nurse in managing students with type 1 diabetes mellitus. The design of the questionnaire was informed by an extensive literature review of previous studies on this topic. In addition, socio-demographic information was collected to profile participants and contextualize their perspectives.

Ethical considerations

All ethical and deontological standards were observed to ensure the study’s validity and reliability. The study was approved by the Department of Nursing of the University of Thessaly Ethics Committee (01/02-09-2024). Participants were informed about the study’s purpose, objectives, procedures, and required time commitment. Written consent was obtained for participation and audio recordings of the interviews, and participants were assured of the confidentiality of their data. To protect anonymity, participants were assigned unique codes, and all audio recordings, transcripts, and analysis files were stored securely on the researcher’s computer, accessible only to the researcher and the supervising professor. Data will be destroyed two years after the study’s conclusion. Participants were informed about their right to withdraw at any time without providing a reason. They were also notified that they could access the study’s findings and any potential publications resulting from the research.

Data analysis

The completion of the recording of the respondents' answers to all the questions of the semi-structured interviews was followed by the transcription of these answers, during which the exact recording of their answers was sought. The transcript of the transcript was subsequently utilized in order to analyze the implemented interviews and extract the corresponding findings thematically. The thematic analysis of the materialised interviews subsequently allowed the understanding of the transcript texts and the identification of the corresponding thematic sections. Using a purely inductive process, efforts were made to group the interviewees' responses and the projected information, allowing their organization within distinct conceptual units, with their subsequent coding and categorization based on the common content of the collected responses [17]. To enhance the reliability of the findings, two researchers independently reviewed and analyzed the data, ensuring consistency in the interpretation and identification of key themes.

## Results

This qualitative study was conducted through semi-structured interviews and included 10 educators employed in General Education structures of Primary Education. Among the participants, the majority were women (n=7, 70%), with only three male participants. Most belonged to the age group of “25-35 years” (n=4, 40%), while two participants were in the “35-45 years” age group, two were in the “45-55 years” age group, and two participants fell into the “55 years and above” category. In terms of educational background, 70% (n=7) of the educators had pursued postgraduate studies, with only three participants (30%) holding just a bachelor’s degree from a higher education institution. Regarding years of professional experience, a total of four educators (40%) had less than 10 years of experience, three educators (30%) had less than 15 years of experience, and three educators had more than 25 years of experience. Detailed characteristics of the participants are presented in Table 1.

**Table 1:** Demographic characteristics of participating educators

Educator	Gender	Age	Education level	Years of teaching experience
Educator A	Female	35-45	Postgraduate degree	15
Educator B	Female	25-35	Postgraduate degree	6
Educator C	Male	45-55	Postgraduate degree	13
Educator D	Female	25-35	Postgraduate degree	8
Educator E	Male	55 and above	Bachelor’s degree	30
Educator F	Female	25-35	Bachelor’s degree	7
Educator G	Female	25-35	Postgraduate degree	8
Educator H	Female	35-45	Postgraduate degree	10
Educator I	Male	55 and above	Postgraduate degree	36
Educator J	Female	45-55	Bachelor’s degree	25

Three main themes emerged from the thematic analysis. The first theme was Knowledge and Understanding of Type 1 Diabetes Mellitus, second was Knowledge and Perceptions of the Role of the School Nurse in Supporting Students With Diabetes, and the third theme was Collaboration and Interaction Levels Between Teachers and the School Nurse. Themes and examples of quotes are presented in Table 2.

**Table 2:** Themes that emerged from the analysis

Themes	Example of quotes
Knowledge and Understanding of Type 1 Diabetes Mellitus: Perceptions and Experiences of Teachers	“…I know that it is essentially an autoimmune disease that primarily affects young individuals at an early age, destroying the beta cells of the pancreas that produce insulin, which makes the patient immediately insulin-dependent….”
Knowledge and Perceptions of Teachers Regarding the Role of the School Nurse in Supporting Students With Type 1 Diabetes	“…I consider the role of the school nurse to be quite multifaceted, but I would say that one of their primary duties is managing children with chronic illnesses, possibly providing first aid, managing certain medications in the school pharmacy, and offering counselling support to both parents and teachers….”
Collaboration and Interaction Levels Between Teachers and the School Nurse in the Management of Students with Type 1 Diabetes	“…Our collaboration was exemplary…. She provided me with security regarding my student’s health…. Her relationship with the other children in the class, as well as with all the students at our school, was one of love and trust….”

Knowledge and understanding of T1DM: perceptions and experiences of teachers

The entire group of participating educators demonstrated adequate awareness regarding the nature and symptoms of type 1 diabetes, with the majority able to provide detailed information about the potential complications of the disease. Participant C specifically noted, “…I know that it is essentially an autoimmune disease that primarily affects young individuals at an early age, destroying the beta cells of the pancreas that produce insulin, which makes the patient immediately insulin-dependent….” Similarly, Participant J provided clear insights into the disease’s pathophysiology and management: “…I know that it is a condition that appears at a young age and has no cure, but it can be managed with insulin injections….” Additionally, Participant B clearly described several symptoms of the disease, demonstrating extensive knowledge in this area: “…I know that it is a chronic autoimmune disease that raises glucose levels…. Another thing I know is the symptoms: frequent urination, excessive thirst, and sometimes weight loss or fatigue….”

Most educators reported being informed about managing cases related to T1DM within the school environment, with information coming from specialized healthcare professionals or nurses. Participant B explained: “…I learned through my collaboration with the school nurse to use the glucose monitoring device that the children use, and sometimes help my student with their glucose measurements and some general aspects of managing the disease, such as diet and exercise….” Participant D expressed confidence in their ability to support a student with diabetes, provided they received the necessary guidance: “…Yes, I have attended in-school training by our school nurse regarding the management of Type 1 Diabetes Mellitus. The training covered normal and abnormal glucose levels, symptoms of hypoglycemia and hyperglycemia, how to handle these episodes based on blood sugar readings, and how to use and administer glucagon injections….” Participant I participated in a seminar aimed at educating educators about the disease and its management: “…Yes, from a training seminar … we learned about symptoms, how to manage them, and potential complications….” Similarly, Participant J attended a similar seminar where a healthcare professional provided specific information about the disease’s symptoms, causes of serious complications, and how to manage them: “…Yes, at one point, a nurse held a seminar on diabetes. He taught us how to recognize symptoms, what to do in emergencies, and even mentioned how children with diabetes may sometimes exhibit mood swings or irritability….”

Knowledge and perceptions of teachers regarding the role of the school nurse in supporting students with T1DM

The entire group of participating teachers appears to be familiar with the concept of the school nurse as well as the full scope of their duties while simultaneously emphasizing the undeniable value of their role within the school community. Specifically, Participant B referred to the multifaceted role of the school nurse: “…I consider the role of the school nurse to be quite multifaceted, but I would say that one of their primary duties is managing children with chronic illnesses, possibly providing first aid, managing certain medications in the school pharmacy, and offering counselling support to both parents and teachers….” Meanwhile, Participant E clearly outlined the duties of the school nurse: “…School nurses belong to the special educational staff, and along with teachers, they are part of the school staff. They are there to look after and care for everyone, both the young and the old!” Participant H also demonstrated a solid understanding of the school nurse’s role: “…Their role is to ensure the physical well-being of the children, administer medications, and be generally present for the children…. Additionally, they can, through health education programs, teach children various things related to their hygiene….” Similarly, Participant A stated: “…I know that, apart from managing emergency situations, the nurse is always close to these children and helps them with their diet because, as we have been informed, their meals should be at specific times to maintain their blood glucose levels within the right range….”

Participant A also highlighted the contribution of the school nurse to ensuring equal participation of diabetic students in the educational process: “…In the past, these children used to miss a lot of school; they wouldn’t come to school at all. However, now, with the presence of the nurse, they are at school every day….” Beyond providing assistance during emergencies, all teachers recognized the value of the psychological and emotional support that the diabetic student receives due to the presence of the school nurse in the school environment. Specifically, Participant A stated, “…The nurse helps the child psychologically and supports their communication … and in interactions with other children, because sometimes these children can become victims of bullying, so the nurse is there to balance things out….” Similarly, Participant E acknowledged the special relationship that develops between the child with diabetes and the school nurse: “…The child often sees the nurse as a family member…. There is mutual trust…. The nurse creates a sense of security for the child with great discretion…. The child understands that, for anything that happens, the nurse’s experience and embrace will always be available….” Considering the overall contribution of the school nurse in managing the diabetic child and handling emergencies within the school, all teachers recognized the value of their presence. Specifically, teachers described the school nurse as “essential,” with Participant B stating, “…They are people who perform a function, not just a job….”

Collaboration and interaction levels between teachers and the school nurse in the management of students with T1DM

Regarding their collaboration with the school nurse, all participating teachers expressed satisfaction, describing the interaction as “excellent.” Specifically, Participant D mentioned, “…My cooperation with the nurse was amazing. She was cooperative and always willing to help with anything….” Participant E presented a particularly positive view, describing the trust that developed between all members of the school community and the nurse: “…Our collaboration was exemplary…. She provided me with security regarding my student’s health…. Her relationship with the other children in the class, as well as with all the students at our school, was one of love and trust….”

In terms of managing the student with T1DM, the collaboration between teachers and the school nurse was described as “excellent” by almost all participants, with each teacher encouraged to express their concerns and fears and receive appropriate support. Specifically, Participant A stated, “…Whenever needed, the school nurse is always available to us….” The frequent and seamless communication between teachers and the school nurse significantly contributed to the optimal monitoring of the diabetic child, as noted by Participant C: “…The communication was frequent, I could say every time, and both the nurse and I would check how things were going….” Participant F also reached out to the school nurse based on the diabetic child’s needs: “…As needed … when I see that the child requires the nurse’s assistance, I will contact her….” Participant B also highlighted the provision of a suitable manual of instructions by the school nurse, aimed at offering optimal support in case of emergencies during her absence, reflecting the high sense of responsibility toward her role: “…She had made sure to leave a manual at the school with very basic initial steps to ensure the teachers felt some security until she could attend the incident in person….”

Recognizing the excellent collaboration with the school nurse and understanding the crucial role she plays, all teachers emphasized the importance of her presence within the school, supporting the need for the establishment of more positions to meet the numerous demands arising within the school environment. Specifically, Participant D remarked, “…I believe school nurses should be in every school to manage chronic diseases like diabetes, but also to assist other children in the school facing issues like accidents…. Hiring school nurses in all schools should become institutionalized…. I sincerely hope for this….”

Participant E advocated for the permanent presence of a school nurse in every school: “…It is essential for all multi-class schools to have the services of a school nurse daily, not just on certain days of the week…. At the same time, the Ministry of Education and Religious Affairs must proceed with mass permanent appointments of school nurses, so that all schools in the country are staffed with nurses….”

## Discussion

The present study aimed to explore the knowledge and experiences of educators regarding the contribution of school nurses in managing and supporting students presenting with symptoms of type 1 diabetes mellitus. Through qualitative research involving primary education teachers, this study sought to elucidate their experiences, focusing on the quality of their relationship with the school nurse and their perceptions of the necessity of employing school nurses in the educational setting. All in all, the participating teachers demonstrated a comprehensive understanding of diabetes mellitus, including its nature, symptomatology, and potential complications. They were well-informed about the role and responsibilities of the school nurse, recognizing the significant contribution of this professional within the school community. Moreover, their cooperation with the school nurse was characterized by a high level of satisfaction, with all teachers describing their collaboration as “excellent.”

Specifically, regarding educators’ knowledge and experiences with managing students with T1DM, all participants demonstrated adequate awareness of the disease’s symptomatology. Most could provide sufficient information on the potential occurrence of severe complications. These findings align with those of similar studies, which also report sufficient awareness among the educational community regarding T1DM, contributing to the optimal management of affected students [3,9,18,19]. Teachers’ adequate knowledge of the symptomatology and management of T1DM is crucial for the safety and well-being of students with the condition. The American Diabetes Association emphasizes that training school staff in diabetes care is essential for effectively managing students’ needs [20]. A lack of such training can contribute to feelings of anxiety and inadequacy among educators. Research has shown that insufficient awareness can lead to stress and concern among teachers, negatively impacting their ability to effectively handle diabetes-related situations [20]. Specifically, a 2020 study by Gutzweiler et al. highlighted three key areas where teachers often report deficiencies: knowledge about diabetes and its management, institutional support, and communication with parents and healthcare professionals [21]. These gaps can result in increased anxiety and insecurity when addressing students with T1DM [21]. Furthermore, inadequate teacher awareness regarding disease management and emerging complications contributes to feelings of stress and concern, as reported by one of the study participants. Similar anxiety about the unsuccessful management of students with T1DM has also been documented in the study by Almehmad et al. [11].

Educators’ sources of information about addressing T1DM-related incidents or complications within the school environment varied. The majority relied on specialized health professionals or nurses for guidance, as also noted by Statiri et al. [12]. Participation in educational seminars was also recognized as a credible source of information, echoing study findings of Alluhaybi et al., where educators expressed a desire to participate in training programs on key issues affecting the school community [8].

In terms of educators’ perceptions of the school nurse’s role in supporting students with T1DM, all participants acknowledged the school nurse as an integral figure and expressed strong support for their role within schools. These findings are consistent with a body of related literature [22-24]. Additionally, all educators appreciated the contribution of school nurses in providing psychosocial support to diabetic students. This aligns with findings from other studies, which emphasize the positive impact of school nurses in ensuring students’ psychosocial well-being [25-27].

The above findings reveal educators’ positive attitudes toward the presence of school nurses in educational settings. All participants underscored the necessity of this institution, a conclusion corroborated by Cangelosi et al. [24]. Furthermore, regarding the collaboration between educators and school nurses in managing students with T1DM, all participants reported satisfaction with their cooperation. The interaction was described as “excellent,” characterized by frequent and seamless communication during the monitoring of diabetic students. These results are further supported by similar studies [26,28], which highlight the significant role of school nurses, whose presence in schools is deemed essential.

At the core of qualitative research approaches lies the ability to delve deeply into the subjective perspectives of the respondents, utilizing semi-structured questions and enabling the development of a dialogical conversation with the researcher. While this approach allows for thorough exploration of the participants’ experiences and perceptions, it also carries the risk of the researcher’s subjective biases being projected, which may divert the study from its original objective. In contrast to quantitative methodologies, which rely on statistical analysis and the objective presentation of results, qualitative research is a more personalized process, requiring the activation of the researcher’s communication skills to ensure the validity of its execution [14,29].

A key stage in the execution of this study and in addressing the research questions was the data collection process through semi-structured interviews. The scheduling of these interviews was based on the availability of the participating educators, with the interviews being conducted within the environment of their school units. While the choice of location significantly contributed to the comfort of the educators due to their familiarity with the setting, it also presented notable limitations. The primary limitation was the inability to find an isolated space suitable for conducting uninterrupted conversations between the researcher and the interviewee. Disruptions from the surrounding environment occasionally interrupted the flow of the discussion, distracting the participant from the process. One more limitation is that the small sample size along with the qualitative nature of the study does not allow generalization of results.

## Conclusions

In conclusion, this study highlights the critical role of school nurses in supporting students with type 1 diabetes mellitus and promoting the health of all members of the school community. The findings underscore the need to increase the presence of school nurses in all schools, focusing on creating a safe environment that allows for equal participation of every student in the learning process. Recognizing the importance of including all students in the educational process, regardless of the health issues they may face and their specific needs, the role of the school nurse emerges as critically important. The school nurse is essential as a key member of the school community, aiming to support students and ensure their health. This qualitative study sought to capture the attitudes and perspectives of educators regarding the school nurse, particularly in managing students with T1DM symptoms.

The findings of this study highlight a unanimous agreement among the participating educators on the urgent need for collaboration with the school nurse in health-related matters, stressing the necessity of having school nurses in all educational institutions. The value of having a specialized healthcare professional in the school setting is clear, as it provides immediate medical care in emergencies and manages potential complications in students with chronic conditions. Establishing effective channels of communication between educators and the school nurse enables a holistic approach to students’ well-being, providing individualized care and support while respecting the unique nature of their condition. Future research should further investigate the effectiveness of diabetes education programs for teachers and explore ways to optimize the role of school nurses in supporting students with chronic conditions.

## Appendices

 Appendix 1

**Table 3:** Interview guide

Question number	Interview guide
1	What is your knowledge regarding type 1 diabetes?
2	What do you know about the nursing profession, and what could be the nurse's role in a school setting?
3	Have you received any information or training on managing type 1 diabetes from a nurse or another healthcare professional? If so, what kind of information?
4	What is your experience as an educator with students who have type 1 diabetes?
5	How often do you communicate with the school nurse regarding students with diabetes?
6	Can you describe a specific incident where the nurse managed a case involving a child with diabetes?
7	Besides handling emergency situations, what other services or support does the nurse provide to students with diabetes?
8	How do you receive support from the nurse in situations where they are not immediately available?
9	How do you assess the contribution of the school nurse to the management of diabetes in your students?
10	How would you describe the effectiveness of your collaboration with the school nurse?
11	Is there anything you would like to see changed or improved in your collaboration with school nurses, or anything you would like to add regarding their role in managing students with type 1 diabetes?
